# HIV and Hepatitis B and C incidence rates in US correctional populations and high risk groups: a systematic review and meta-analysis

**DOI:** 10.1186/1471-2458-10-777

**Published:** 2010-12-21

**Authors:** Ethan Gough, Mirjam C Kempf, Laura Graham, Marvin Manzanero, Edward W Hook, Al Bartolucci, Eric Chamot

**Affiliations:** 1 Epidemiology Unit, Ministry of Health, Belmopan City, Belize; 2 Department of Epidemiology, University of Alabama at Birmingham, Birmingham, Alabama, USA; 3 National AIDS Program, Ministry of Health, Belmopan City, Belize; 4 Department of Medicine, University of Alabama at Birmingham, Birmingham, Alabama, USA; 5 Department of Biostatistics, University of Alabama at Birmingham, Birmingham, Alabama, USA

## Abstract

**Background:**

High Human Immunodeficiency Virus (HIV) prevalence and high risk behaviors have been well documented within United States (US) correctional systems. However, uncertainty remains regarding the extent to which placing people in prison or jail increases their risk of HIV infection, and regarding which inmate populations experience an increased incidence of HIV. Describing these dynamics more clearly is essential to understanding how inmates and former detainees may be a source for further spread of HIV to the general US population.

**Methods:**

The authors conducted a systematic review and meta-analysis of studies describing HIV incidence in US correctional facility residents and, for comparison, in high risk groups for HIV infection, such as non-incarcerated intravenous drug users (IVDU) and men who have sex with men (MSM) in the US. HIV incidence rates were further compared with Hepatitis B and Hepatitis C Virus rates in these same populations.

**Results:**

Thirty-six predominantly prospective cohort studies were included. Across all infection outcomes, continuously incarcerated inmates and treatment recruited IVDU showed the lowest incidence, while MSM and street recruited IVDU showed the highest. HIV incidence was highest among inmates released and re-incarcerated. Possible sources of heterogeneity identified among HIV studies were risk population and race.

**Conclusions:**

Although important literature gaps were found, current evidence suggests that policies and interventions for HIV prevention in correctional populations should prioritize curtailing risk of infection during the post-release period. Future research should evaluate HIV incidence rates in inmate populations, accounting for proportion of high risk sub-groups.

## Background

In 2008, nearly 2.4 million people were incarcerated in United States (US) jails or prisons [1,2]. Furthermore, about 25% of persons living with the Human Immunodeficiency virus (HIV) and about 30% of those living with Hepatitis C Virus (HCV) infection spent time in correctional facilities [3]. As a result, many have expressed concerns that transmission of blood-borne infections among inmates may be a major source for further spread to the general population [4-7].

Three lines of evidence support this view: (1) the prevalence of HIV infection, viral hepatitis, and sexually-transmitted infections (STI) is typically higher in incarcerated than in non-incarcerated populations; (2) illicit drug injection, unprotected sexual activity, and other risky behaviors are common in prisons and jails; and (3) correlative studies have repeatedly found independent associations between antecedents of incarceration and increased risk for infections such as HIV.

In more details, despite declines since the 1990s, it has been estimated in 2008 that 1.5% of the total US custody population in federal and state prisons was infected with HIV [8]; that 12.0% to 35.0% had chronic HCV infection [3]; and that 1.0% to 3.7% had serological markers of chronic HBV infection [3]. Based on these estimates, the prevalence of HIV was about four times higher among prison inmates than in the general population [9]. Likewise, the prevalence of chronic HBV infection was 2 to 6 times higher, and that of chronic HCV infection was up to 10 times higher, among prison inmates than in the community.

In contrast, more uncertainty remains about the extent of high-risk behavior taking place within US correctional facilities. Due to important differences between institutions in enabling factors such as overcrowding and understaffing, estimates of interest vary widely. Recent studies suggest that 3.0% to 28.0% of adult inmates use intravenous drugs while incarcerated; 4.0% to 65.0% engage in unprotected homosexual activities [3,10,11]; and 0.0% to 15.7% report sexual victimization during incarceration [12]. Prison entrants, incarcerated inmates, and intravenous drug users (IVDU) also tend to share a number of incarceration-related factors that predict HIV infection, including overall length of time spent incarcerated [13], repeated incarceration [14], tattooing in prison [15-17], and history of syringe sharing in prison [18-20]. Comparable observations have been made for other blood-borne infections and STI [14,21-26].

As just discussed, high HIV prevalence and high risk behavior within correctional systems are well documented. There is much less evidence, however, to support the notion that the correctional setting increases the incidence of HIV, and thus plays a central role in sustaining or increasing community rates when inmates are released. Many investigators, in fact, have suggested that inmates appear more likely to acquire infection outside than inside correctional facilities [3,10,27]. Since determining the role of incarceration in the epidemiology of HIV transmission is a crucial step toward formulating cost-effective public health policies and interventions for US HIV/AIDS control, we conducted a systematic and comparative literature review of HIV, HBV, and HCV incidence among residents of correctional facilities, released detainees, community-living IVDU and community-living men who have sex with men (MSM) in the US. Our aims were twofold: to summarize the published literature on HIV incidence rates and other key blood-borne infections, such as HBV and HCV, in US correctional facilities; and to compare these with infection rates among non-incarcerated individuals who bear high burdens of blood-borne infections and STIs. We hypothesized that inmates experience a lower incidence of HIV than community living risk groups that practice the behaviors which place incarcerated populations at increased risk. We further postulated that comparisons between viruses that share the same routes of transmission in prisons, jails, and high risk groups in the community will provide the comparative basis to more clearly elucidate the role the correctional setting may play in increased risk of infection and the likely avenues for further spread to the community.

## Methods

### Search Strategy and Selection Criteria

The authors searched Medline/PubMed, PsycINFO/EbscoHost and Embase/Scopus (January 1990 to September 2009) for English language studies conducted in the US. Medline searches used permutations of medical subject headings (MeSH) and subheadings for each risk group and infection outcome of interest. In Embase, searches were developed using Emtrees and author defined keywords for relevant articles identified through the Medline searches. The same keyword strategy was used with PsycINFO (Table 1). References of all review articles identified in the search [3,7,10,27-32] and of all articles selected for full review were hand-searched for additional studies. All search strings were developed with the assistance of a qualified librarian.

**Table 1:** Search Strategies

Search #	Medline	PsychInfo	Scopus
1	(prisons OR prisoners) AND (HIV infections/transmission OR HIV infections/epidemiology OR HIV infections/prevention and control)	(human immunodeficiency virus OR acquired immune deficiency syndrome) AND (prisoners OR prisons)	HIV AND prison* AND transmission
2	(prisons OR prisoners) AND (substance abuse, intravenous OR needle sharing OR tattooing)	(incarceration OR institutional schools OR maximum security facilities OR correctional institutions OR prisons OR reformatories) AND (human immunodeficiency virus OR acquired immune deficiency syndrome)	HIV and (prison* OR inmate*) AND (injection drug OR intravenous drug)
3	(prisons OR prisoners) AND (hepatitis c OR hepatitis c virus OR hepatitis b OR hepatitis b virus)	1 AND (cohort analysis OR longitudinal studies) AND male homosexuality	(hepatitis c OR hepatitis B) AND (incidence OR epidemiology OR cohort) AND (United States) AND (homosexual* W/3 male OR men who have sex with men)
4	3 AND homosexuality	(intravenous drug usage OR needle sharing) AND (cohort analysis OR longitudinal studies) AND male homosexuality	(incidence OR epidemiology OR cohort) AND (United States) AND (needle W/3 sharing OR intravenous W/5 substance *use* OR intravenous W/5 drug *use* OR ivdu) AND (hepatitis c OR hepatitis b)
5	(HIV infections/transmission OR HIV infections/epidemiology) AND (incidence OR cohort studies) AND (United States) AND (homosexuality, male)	(male homosexuality AND (epidemiology OR cohort analysis OR longitudinal studies) AND (hepatitis)	(hiv OR aids OR human immunodeficiency virus OR acquired immune deficiency syndrome) AND (incidence OR epidemiology OR cohort) AND (United States) AND (needle W/3 sharing OR intravenous W/5 substance *use*) OR intravenous W/5 drug *use* OR ivdu)
6	5 (NOT homosexuality, male) AND (substance abuse, intravenous OR needle sharing)	(intravenous drug usage OR needle sharing) AND (epidemiology OR cohort analysis OR longitudinal studies) AND (hepatitis)	(United States) AND (incidence OR cohort OR epidemiology) AND (hiv OR aids OR human immunodeficiency virus OR acquired immune deficiency syndrome) AND (homosexual* W/5 male OR men have sex with men)
7	(hepatitis c OR hepatitis c virus OR hepatitis b OR hepatitis b virus) AND (homosexuality, male) AND (United States) AND (incidence OR cohort studies OR epidemiology)		
8	7 (NOT homosexuality, male) AND (substance abuse, intravenous OR needle sharing)		

Search terms used during the literature search are reproduced here. Each search was numbered, and numbers indicate where searches were combined to create more specific search criteria. Abbreviations: HIV, Human Immunodeficiency Virus.

Two investigators (EG and LG) independently assessed titles and abstracts to identify original research studies eligible for review. If eligibility could not be determined from reviewing titles and abstracts, the full article was retrieved. An article was chosen if it reported an incidence density (or cumulative incidence per year at risk) for one or more infections of interest (HIV, HBV, HCV) among inmates (incarcerated, released, reincarcerated), non-incarcerated high-risk individuals (MSM, IVDU) or both. Articles that did not provide original data, case reports, legal cases, case-control studies, and reports of outbreak investigations were excluded. We also excluded estimates of infection incidence measured among individuals entering the prison system for the first time, since these individuals had not been exposed to the correctional environment yet and, therefore, were representative of their community of origin rather than of the inmate population.

### Data Abstraction

Once eligibility was determined, two reviewers (EG and MM) independently extracted data from selected articles using a standardized checklist. Discrepancies were corrected by consensus. For each infection and population of interest, the following information was retrieved: sample characteristics (age, sex and race composition, number of subjects at risk, and prevalence of infection at baseline), risk behaviors (same sex risk behaviors, intravenous drug use), number of infections during follow-up, length of follow-up, and attrition rate. Information on study characteristics was also collected (study period, site of data collection, study design, diagnostic methods, and study limitations). Depending on data availability, data on demographic characteristics and risk behavior described either the total sample tested at baseline or the baseline seronegative sample. Corresponding authors were contacted by email for data of interest not published in the articles. Of the authors contacted, 34.0% (12/34) responded with the data requested, 27.0% (9/34) responded saying that data were no longer available, and the remainder did not respond. When multiple publications reported on the same study cohort, we used the most recent and complete data.

### Operational definitions

*Baseline prevalence of infection *was defined as the percentage of subjects who tested positive for infection at baseline; *incidence density of infection *as the average number of new infections in baseline seronegative subjects per 100 person-years (py) of follow-up; *cumulative incidence of infection *as the percentage of subjects diagnosed with a new infection among baseline seronegative subjects who had ≥1 follow-up test; *attrition rate *as the percentage of baseline seronegative subjects who did not undergo ≥1 follow-up; and *predominant race *(*sex*) as the ethnic or racial group (sex) that comprised the majority of study subjects. *Continuously incarcerated inmate populations (CIIP) *were defined as cohorts of inmates tested at entry, or post entry, with follow-up after at least 12 months of incarceration. There was one exception in which inmates were tested at entry or post entry, with follow-up testing at exit, regardless of the duration of their incarceration [33]. *Inmates released and reincarcerated (IRAR) *were defined as cohorts of inmates with at least two incarcerations during the study period who were tested at each intake, booking, or time of incarceration, with the exception of one study in which the authors described the cohort as "reincarcerated" [34].

### Statistical analysis

Where person-years of follow-up and median follow-up time were reported neither by the article nor by the contacted author, we estimated total person-years accrued from the reported incidence per 100py and the total number of new infections (13 articles). For two studies [35,36], incidence density was calculated from data on cumulative incidence and median follow-up time. To ensure comparability of confidence limits across studies regardless of sample size, 95% confidence intervals (95%CI) were recalculated for all incidence density estimates using the exact Poisson method [37].

Due to significant heterogeneity among studies demonstrated in a fixed effects model, pooled estimates of incidence density and 95%CI were obtained for each infection and population of interest using the DerSimonian-Laird random effects method [38]. Where the number of incident cases was zero, a value of 0.5 was assigned in order to estimate standard error for pooled incidence. Heterogeneity among studies was assessed using the *I*^2 ^statistic, which estimates the proportion of total variation that is due to heterogeneity beyond chance [39]. Publication bias was assessed using Egger's test [40].

For each infection of interest, we calculated separate pooled estimates for HIV, HBV and HCV incidence among continuously incarcerated inmates and inmates released and re-incarcerated. Similarly, we calculated separate pooled incidence estimates for IVDU recruited through street outreach, IVDU recruited from drug treatment programs or clinics, IVDU recruited using either approach, and all categories of IVDU combined. To explore potential sources of heterogeneity, we conducted a random-effects meta-regression analysis of HIV studies only (the number of published estimates of HCV and HBV incidence was too small to warrant separate meta-regression analyses). The potential sources of inter-study variability were defined a priori and included: sample size, risk population, percent IVDU, percent MSM, mean age, predominant race and sex, geographic location, attrition rate, baseline prevalence, person years of follow-up, and study start year. We also defined potential study design characteristics that could be sources of heterogeneity or bias as suggested by the MOOSE statement [41]. These included publication year and follow-up design (prospective or retrospective). Finally, we included whether authors had been contacted for unpublished data, and whether they responded, as two proxy variables for the completeness and availability of study data.

A sensitivity analysis was conducted to determine the robustness of HIV incidence results to the inclusion/exclusion of studies that provided incomplete or imprecise HIV incidence data. All analyses were conducted using *SAS version 9.2 *(SAS Institute, Cary, NC) and Microsoft Excel XP (Microsoft Corp., Redmond, Washington, USA).

## Results

### Literature Review

**Figure 1:**
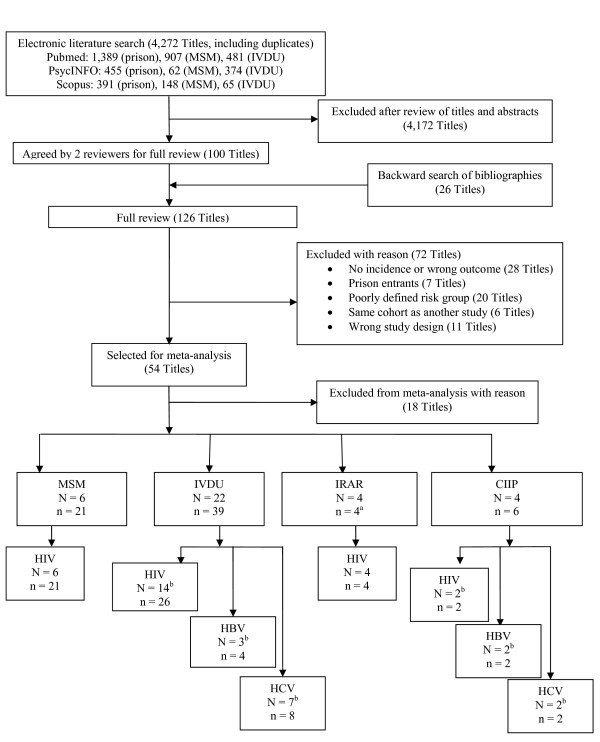
**Flow Diagram of Study Selection**. CIIP, continuously incarcerated inmate population; IRAR, inmates released and reincarcerated; IVDU, intravenous drug users; MSM, men who have sex with men; N, number of articles included in the meta-analysis; n, number of incidence estimates included in the meta-analysis; ^a^The number of articles and incidence estimates do not add up because one IVDU title included an IRAR site; ^b^The number of articles included for each outcome do not add up to the total number of articles in each risk group because three titles included >1 outcome.

The electronic search identified 4,272 titles, of which 4,172 were excluded based on review of titles and abstracts (Figure 1). Full text was retrieved for the remaining 100. A backward search of references identified 26 additional titles resulting in 126 articles selected for full review. Of these, 72 were excluded based on study eligibility criteria and an additional 18 were excluded because they provided insufficient information to calculate exact 95%CI and standard errors for meta-analysis [42-47]; did not report annualized incidence or median follow-up time [48-54]; provided potentially biased estimates of HIV incidence based on a self-reported date of last seronegative test [55-58]; or calculated incidence estimates using a mathematical model [58]. Additionally, data from two locations (Los Angeles and San Jose, California) from one multisite study were excluded because insufficient information was provided to calculate standard errors for meta-analysis [34].

### Study Characteristics

In total 36 unique studies were included in the meta-analysis (Additional file 1) [33-36,60-91]. Numerical data of interest were often difficult to locate (median Kappa statistic of agreement among reviewers for identification of six key variables, 0.46). In contrast, inter-rater agreement on abstracted data was high once the information was found (median intra-class coefficient of 1.0 for same six key variables). Of the 36 studies, 10 reported an incidence from more than one geographic location [34,64,69-71,75,77,78,81,82]. Four studies reported one or more incidence estimates for continuously incarcerated inmates [34,35,60,83], four for inmates released and reincarcerated [34,36,61,62], 23 for IVDU [34,63-76,84-91], and six for MSM [77-82]. Together, these studies yielded 53 estimates of HIV incidence, 10 estimates of HCV incidence, and 6 estimates of HBV incidence (Figure 1). For HIV, data were found on 2 seroconversions in 1,901py of follow-up among continuously incarcerated inmates, 101 seroconversions in 5,253py of follow-up among inmates released and reincarcerated, 650 seroconversions in 37,137py among IVDU, and 777 in 33,096py among MSM. HCV studies reported 4 seroconversions in 733py among continuously incarcerated inmates and 305 seroconversions in 2,544py among IVDU. Finally, HBV studies reported 33 seroconversions in 1,970py of follow-up among continuously incarcerated inmates, and 153 seroconversions in 1,193py among IVDU.

The majority of studies reviewed were prospective cohort studies (86.1%) (Additional file 1). Retrospective studies included cohorts of inmates with stored specimens from testing at admission [61,62,83], and cohorts of IVDU with stored specimens from repeat testing at drug treatment clinics [75,76]. Inmate and MSM cohorts were predominantly white (40.4%-85.8%), while IVDU cohorts were predominantly African American (41.0%-93.3%) (Additional file 2). Cohorts of continuously incarcerated inmates included 94.0%-100.0% of men, whereas cohorts of inmates released and reincarcerated were composed of either men [36] or women [34,61,62] exclusively; cohorts of IVDU included 50.0% to 80.0% of men.

For all infection outcomes and populations, the modal start year for data collection was 1994 (range 1984 to 2000). Most inmate studies began data collection in 1985 (range 1985 to 2000), while most IVDU and MSM studies began data collection later, i.e., in 1994 (range 1985 to 2000) and 1995 (range 1984 to 1999) respectively (Additional file 2).

Mean HIV baseline prevalence was 2.1% in continuously incarcerated inmates (n = 2), 6.4% in inmates released and reincarcerated (n = 4), 18.5% among all IVDU studies (n = 16), and 6.1% among MSM studies (n = 1). HCV baseline prevalence was 30.6% among continuously incarcerated inmates (n = 2) and 52.4% among all IVDU studies (n = 8). Finally, HBV baseline prevalence was 20.3% among continuously incarcerated inmates (n = 2) and 26.7% among all IVDU studies (n = 2) (Additional file 2).

The mean attrition rate was calculated to be 26.4% across all infection outcomes and risk populations. In inmate populations, the mean attrition rate was 19.0% (n = 6); among all IVDU studies it was 40.0% (n = 31), and among MSM studies it was 4.5% (n = 17) (Additional file 2).

In CIIP cohorts, the period of incarceration was at least 12 months [35,60,83], with one study reporting 8.5 years as the median [35]. Horsburgh et al. reported the incarceration period for the 2 seroconverters identified only (20 and 130 days) [33]. In IRAR cohorts, the periods of incarceration before release were reported as a mean of 4 days [61], mean 62 days [62], as "days following their arrest" [34], or were not reported [36]. The periods between incarcerations on the other hand were reported as a median of 316 days [36], median of 527 days [62], or were not reported [34,61].

Overall, the most common limitation reported across all risk groups and infections of interest was limited generalizability of study results due to non-random sampling methodology or differential loss to follow-up (Additional file 1).

### Meta-analysis

**Figure 2:**
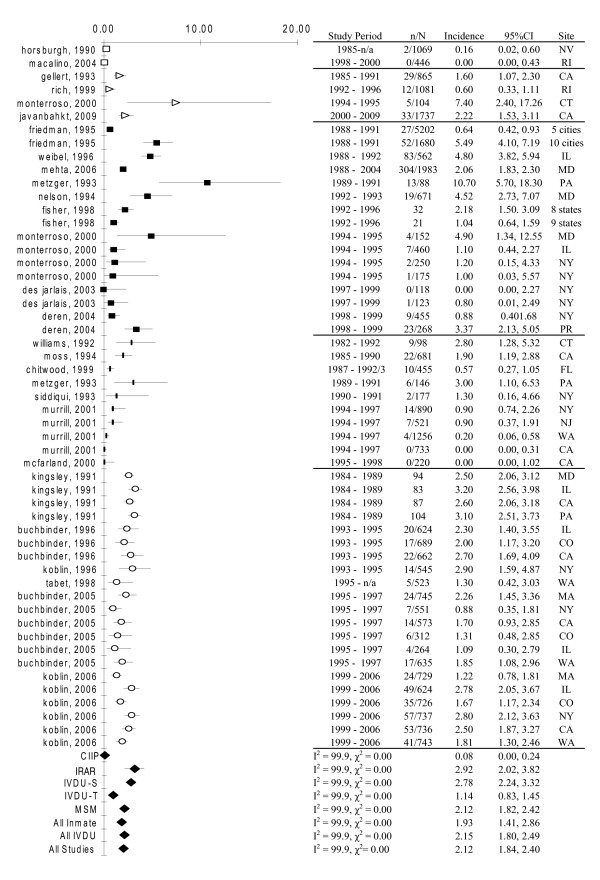
**Meta-analyses of HIV incidence studies**. 95%CI, 95% confidence interval; *I^2^*, I^2 ^statistic; *χ*^2^, chi-squared statistic. ^a^Estimated from cumulative incidence (cumulative incidence = 1-exp(incidence density × time)). White square: CIIP, continuously incarcerated inmate population; White triangle: IRAR, inmates released and reincarcerated; Black square: IVDU-S, intravenous drug users recruited through street outreach; Black vertical bar: IVDU-T, intravenous drug users recruited from drug treatment programs or clinics; White disk: MSM, men who have sex with men; Black diamond: Random effects meta-analysis. Multiple data points from a single study denote incidence estimates from different recruitment sites in multisite studies.

**Figure 3:**
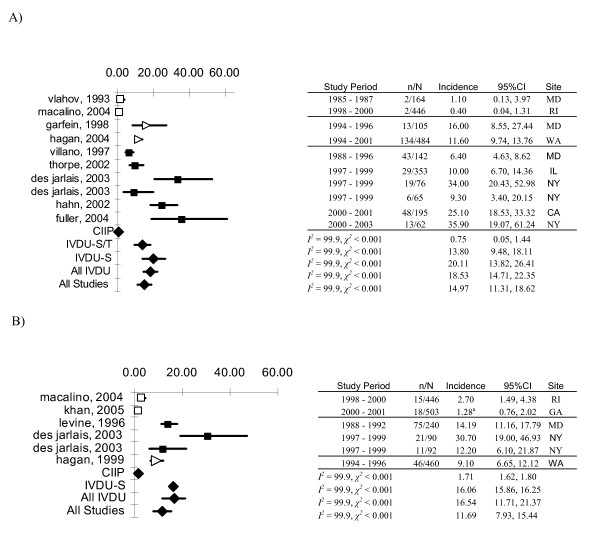
**Meta-analyses of A) HCV Incidence Studies and B) HBV Incidence Studies**. 95%CI, 95% confidence interval; *I^2^*, I^2 ^statistic; *χ^2^*, chi-squared statistic. ^a^Estimated from cumulative incidence (cumulative incidence = 1 - exp(-incidence density × time)). White square: CIIP, continuously incarcerated inmate population; White triangle: IVDUS/T, intravenous drug users recruited through either street outreach or from drug treatment programs or clinics; Black square: IVDU-S, intravenous drug users recruited through street outreach; White disk: MSM, men who have sex with men; Black diamond: Random effects meta-analysis. Multiple data points from a single study denote incidence estimates from different recruitment sites in multisite studies.

The pooled estimate of HIV incidence density was lowest among continuously incarcerated inmates (0.08/100py, 95%CI:0.0,0.24), followed by a more than 10-fold higher incidence for IVDU populations recruited from treatment programs (1.14/100py, 95%CI: 0.83,1.45), and highest among MSM (2.12/100py, 95%CI:1.82,2.42), street recruited IVDU (2.78/100py, 95%CI:2.24,3.32), and inmates released and reincarcerated (2.92/100py, 95%CI:2.02,3.82). Comparison of the 95%CIs suggested that HIV incidence rates were significantly lower among continuously incarcerated inmates and treatment recruited IVDU compared to incidence rates in the other three populations (Figure 2.) Likewise, the pooled estimate of HCV incidence density was lowest among continuously incarcerated inmates (0.75/100py, 95%CI:0.05,1.44) compared to IVDU recruited through a combination of treatment programs and street outreach (13.8/100py, 95%CI:9.48,18.11), and to IVDU recruited exclusively on the street (20.11/100py, 95%CI:13.82,26.41) (Figure 3). Finally, the pooled estimate of HBV incidence density was low among continuously incarcerated inmates (1.71/100py, 95%CI:1.62,1.80) and significantly higher in street recruited IVDU (16.06/100py, 95%CI:15.86,16.25) and all categories of IVDU combined (16.54/100py, 95%CI:11.71,21.37) (Figure 3).

Inspection of forest plots and *I*^2 ^statistics confirmed that there was a high degree of heterogeneity in incidence rates of any given infection across populations; and in incidence of a given infection in a given population across studies (Figure 2, 3). Results of Egger's test suggested that publication bias was present (2-sided *P *= 0.001).

### Meta-regression analyses

After exclusion of sex composition from the list of covariates (because of marked collinearity with the risk population variable), the HIV sub-group meta-regression model explained 52.0% of the variance in pooled incidence of HIV infection. Differences in pooled incidence of infection were independently associated with risk population (2-sided *P *= 0.03), with predominant race (2-sided *P *= 0.03), and with person years of follow-up (2-sided *P *= 0.03), (*data not shown*).

In the model, pooled incidence density of infection was lowest among continuously incarcerated inmates (0.08/100py) and IVDU populations recruited from treatment programs (0.98/100py), followed by MSM (2.12/100py). Higher pooled incidence density of infection were observed among street recruited IVDU populations (2.64/100py; based on 17 published estimates), and inmates who were released and reincarcerated (2.95/100py; based on 4 published estimates). Pooled incidence of infection was higher in study samples that predominantly included African Americans (3.05/100py) compared to Whites (1.79/100py) and other races or ethnicities (1.49/100py) (*data not shown*). Study design and data quality variables included in the meta-regression analysis as previously described were not statistically significant.

### Sensitivity analyses

The sensitivity analyses indicated that HIV incidence results were generally robust to the exclusion of studies discarded from consideration in the main meta-analysis. When excluded articles were included in the analysis [4-44,46,47,57,58,92-94], all pooled estimates of HIV incidence remained within 8.0% of the main meta-analysis results, with a few exceptions. Pooled HIV incidence rates increased for treatment recruited IVDU, (3.31/100py; 95%CI:3.0,3.6), and for all IVDU, (3.06/100py; 95%CI:2.7,3.4), when two of the studies that inferred HIV incidence based on self-reported date of last seronegative test were included [55,56]. These estimates were 2.9 and 1.4 times greater than the estimates from the main analysis for these risk groups. Likewise, pooled HIV incidence rates increased for MSM, (3.45/100py; 95%CI: 3.1,3.8), when two retrospective studies that used stored specimens from routine testing of MSM with primary or secondary syphilis at STD clinics were included [95,96]. This estimate was 1.6 times greater than the estimate from the main analysis for MSM.

## Discussion

Fueled by reports of HIV and STI outbreaks in correctional facilities in the US [11,50,97-101] and in other high-income countries (Scotland [102,103], Australia [17]), the debate about the magnitude of inmate-to-inmate transmission of HIV in the US has spanned more than two decades. The pattern of results that emerged from our comprehensive review and meta-analysis of HIV, HCV, and HBV incidence studies support the notion that the transmission of HIV and other blood-borne infections in US correctional populations occurs at alarmingly high rates during the periods that recidivists spend outside prison. In our study, HIV incidence among inmates released and reincarcerated was much greater (2.92/100py) than in the US general population (0.02 per 100 population in 2006) [104], while HIV incidence among inmates continuously incarcerated was more similar to the general populace (0.08/100py). Rates of HIV seroconversion among reincarcerated inmates were comparable to those typically observed among non-incarcerated individuals who engage in high-risk injecting and sexual behaviors (street-recruited IVDU, 2.78/100py; MSM, 2.12/100py). HIV incidence among IVDU enrolled in a drug treatment program (1.14/100py) laid between the lower bound observed in the general population and the higher bound observed in the high-risk groups. In contrast, intraprison incidences of HCV (0.75/100py) and HBV (1.71/100py) infection were also higher than in the US general population (0.01 and 0.02 per 100 population, respectively, in 2006) [105], but several-fold lower than among non-incarcerated IVDU (HCV, 18.53/100py; HBV, 16.54/100py) and MSM (HCV, no data available; HBV, 15.9%) [51]. The greater incidence of these infections compared with HIV probably reflects the higher prevalence rates of HCV and HBV infection among prison entrants [106] and higher infectivity of HCV and HBV compared with HIV [107,108].

Despite significant heterogeneity among included studies, our results were consistent across meta-analyses and multiple meta-regression analyses. Sensitivity analyses indicated that results were only sensitive to the exclusion of four studies, two in which incidence was inferred from self reported data [55,56], and two in which MSM with early syphilis infection were tested retrospectively [95,96]. Furthermore, our summary estimates of HIV incidence among inmates were comparable with estimates that were published before 1990 [109-112]; did not report data in the desired format [48]; and with studies conducted in Europe [113-115].

Low incidence rates of HIV transmission in prison (range, 0.0 to 0.4/100py) have been reported in three US studies published in the late 1980s [109-111]. The extent to which these older studies further our understanding of the current dynamic of HIV transmission in prison is unclear, since HIV prevalence at prison intake was considerably lower in the 1980s compared with the 2000s. Of note however, study start year and publication year did not significantly contribute to the meta-regression analysis model, suggesting that calendar time was not an important source of heterogeneity among HIV studies. Also, in a US-based study that did not provide annualized incidence density estimates of HIV infection, but retrospectively followed 5,265 male inmates from their entry into custody in 1978 until 2000, 0.63% of the detainees were diagnosed with HIV infection during incarceration and 4.6% after release from prison [48]. Bias may have inflated the difference in incidence during the incarceration and released periods in this study, but no other source of information was found that estimated HIV incidence among inmates released from a US prison as compared to inmates undergoing periods of continuous incarceration.

In three European studies, HIV incidence among detainees ranged between 0 and 1.0/100py [113-115], and was highest among male and female IVDUs recruited in 1987-1988 at a prison remand centre in Sweden (the authors of these studies did not clearly indicate what percentage of inmates had been continuously incarcerated) [115]. In a small Australian study (n = 90), including inmates of both genders, a higher incidence of HCV seroconversion was found among inmates who underwent a period of release before reincarceration compared with inmates who had been continuously incarcerated (10.8 vs. 4.5/100py; *P *= 0.07) [116]. Although the results from these developed nations and from the US seem to be consistent in their documentation of low HIV incidence rates within the prison system and higher rates during post release, international comparisons should be made with great caution given the differences in correctional systems and epidemiological contexts across countries [117]. Hence, two studies identified from the developing world reported higher HIV incidence rates - Brazil (2.8/100py) [118] and Thailand (4.18/100py) [16]. It is clear that an important gap still exists in our understanding of HIV and blood-borne infection transmission in correctional environments throughout the world.

Among MSM, our pooled estimate of HIV incidence is similar to that calculated in a recent study which used a fixed-effects model to calculate a weighted average (2.39%) [119]. In another study, estimates calculated for MSM (0.7/100py) and IVDU (1.5/100/py) differed in comparison to our pooled estimates [120], but this citation [120] did not use meta-analysis methods to estimate HIV incidence in these risk groups.

Our findings are consistent with studies of risky behaviors in correctional populations and hypotheses proposed to explain the apparent paradox of low incidences of HIV, HBV and HCV in the prison system, and high incidence of HIV, HBV and HCV during the post-release periods. As mentioned earlier, the US correctional system offers conditions seemingly favorable to the transmission of blood-borne viruses. There is a large reservoir of potential transmitters in the prison system at any time, and many inmates engage in sexual and drug-mediated risk behaviors regardless of the general lack of condoms [49,50] and sterile injection material [10,48]. For instance, although the frequency of drug use in prison is typically lower than in the general community [48,113,121], there is clear evidence that incarcerated drug users often continue to inject; that injecting equipment is frequently shared among inmates; and that the risk of equipment contamination by parenterally-transmitted viruses is higher within the prison system than outside of it [20,113].

Thus, a possible explanation for the low transmission of HIV, HCV, and HBV within the prison system is that inmates' risk networks are on average considerably smaller and more closed within correctional facilities than in the community. Given the de facto segregation of detainees by age, sex, race, category of offense and, historically in some states, by HIV status [10], it is plausible that the lack of bridges between intra-prison networks, and the small size of the networks, lead to the rapid saturation of the susceptible inmates who have effective contacts with a transmitter [106]. However, the formation of bridges between inmates' risk networks when an adequate proportion of susceptible inmates exists may lead to efficient infection transmission [52,99]. In contrast, studies have shown that many inmates, following re-entry in the community, revert to pre-incarceration habits and engage in high rates of unsafe sexual and intravenous drug use behaviors [122-126], as suggested, for instance, by high frequency of anal sex reporting [124], excess occurrence of drug overdose [125-127], and high risk for mortality [80,128-130] at post release. Among the four studies on inmates released and reincarcerated, those reporting the highest post-release incidence rates followed recidivist female IVDU [34] and recidivist MSM [36]. As such, sub-populations of inmates with risky pre-incarceration behaviors may be at particularly high risk during periods between release and reincarceration.

There was a notable difference in the reported proportion of IVDU and MSM in recidivist inmate populations [34,36,53,62] as compared to continuous inmates [34,35,60,83]. Three of the four recidivist studies reported the proportion of IVDU (59.1% and 100%) or MSM (0.0% and 100%); while two continuous inmate studies reported the proportion of IVDU (2.9% and 11%) or MSM (0.0% and 4.5%) (Additional file 2). These data suggest that the proportion of IVDU and MSM might be larger in recidivist studies than in continuous inmate studies. If real, such a difference would be another explanation for the observed differences in incidence among the studies in continuous inmates, recidivists and community-living populations.

A meta-analysis of pooled incidence rates stratified by risk behaviors would have further clarified whether the recidivist groups are at increased risk of infection compared to their continuous inmate or community living counterparts. However, available data were not sufficient for this type of analysis. Only one study reported the incidence of HBV (8.2/100py) and HCV (5.5/100py) among continuously incarcerated inmates who reported injection drug use [60]. For both infections, the incidence in this group of inmates was greater than the pooled estimate for CIIP cohorts, but was lower than the pooled estimate for community IVDU; the study did not report whether injection drug use occurred during or prior to incarceration.

Our study is subject to limitations. Despite the crucial importance of characterizing the relations between incarceration and the HIV epidemic in the US, we found only five incidence studies published between 1990 and 2004, and one published between 2005 and 2009, that reported primary data on the transmission of HIV in US correctional populations. The recent literature on incidence of blood-borne infections among MSM was also sparse. The importance of further studies on these outcomes in these populations cannot be over emphasized. Our observation of high infection rates among inmates who were released and reincarcerated was based on four studies only and incidence of HIV was relatively low in two of these studies (Figure 2). Three studies evaluated predominantly IVDU women only; one evaluated MSM only; none measured HBV and HCV incidence; and none included inmates who were not reincarcerated after their release.

In the random effects analysis, the limited number of studies that assessed incidence of HIV, HBV or HCV infection made it difficult to disaggregate the *sources *of heterogeneity across studies. Several studies lacked data for inclusion in the meta-regression models and, in general, data were insufficient to properly evaluate the influence of key cofactors, such as sex, age, race, and interaction between population and proportions of subjects engaging in risk behaviors. Other unmeasured factors may also have contributed to the observed heterogeneity.

Egger's test suggested that publication bias might have affected our results. Possible sources of publication bias include citation bias, poor methodological quality of smaller studies, and true heterogeneity. Although study selection criteria were clearly defined and study selection was done by two independent reviewers, we cannot entirely exclude the possibility that some studies were missed due to low citation frequency. Most reviewed studies, in particular small studies, shared one or several important methodological shortcomings, including purposive or convenience samples, inconsistent operational definition of risk populations, short follow-up times, high attrition rates, and inappropriate periods of risk assessment. As already indicated, there was significant heterogeneity across included studies, part of which is likely to have been true heterogeneity. Finally, Egger's test can be sensitive to extreme observations and large sample size, both of which were present in this meta-analysis.

## Conclusion

Our findings support the notion that comprehensive strategies are needed to control the spread of parenterally and sexually transmitted viruses in US correctional populations. Examples are prevention programs to reduce transmission within prison systems [129]; transition programs to better prepare inmates for life after discharge; and interventions to ensure continuity of care in the community. While incarceration does not appear to increase the risk for HIV or other blood-borne disease infection for the average inmate, and while rates of transmission in US correctional settings appear to be lower than would be expected outside prison or jail, the lack of sufficient data for meta-regression and sub-group analyses made it difficult to draw definitive conclusions about the increased risk incarceration poses on high risk groups such as MSM and IVDU, or about the increased risk to these groups and the community during periods of release. In addition to supporting innovative intervention studies, our findings also point to the need for further research to update our understanding of the transmission of blood-borne and sexually-transmitted infections in inmate populations, and, most importantly, to clarify the role of the post release period in infection risk and further spread to the general community. The consistency of our results across infectious agents confirms that HBV and HCV infections may be used as sentinel indicators of risk for HIV infection in correctional settings.

## Competing interests

The authors declare that they have no competing interests.

## Authors' contributions

EG designed the study; coordinated its implementation; performed the literature search, selection of articles, data acquisition, data analysis and interpretation; participated in drafting all sections of the article; and gave final approval of the version to be published. MCK designed the study; helped coordinate its implementation, assisted with the review of relevant literature; helped in data interpretation; participated in drafting all sections of the article; revised the article critically for important intellectual content; and gave final approval of the version to be published. LG performed the literature search and selection of articles; participated in reviewing the manuscript; and gave final approval of the version to be published. MM performed the systematic literature review and data acquisition; participated in reviewing the manuscript; and gave final approval of the version to be published. EWH helped with data interpretation; revised the article critically for important intellectual content; and gave final approval of the version to be published. AB performed data analysis; reviewed the manuscript; and gave final approval of the version to be published. EC designed the study; helped coordinate its implementation; assisted with the review of relevant literature, helped in data interpretation; participated in drafting all sections of the article; revised the article critically for important intellectual content; and gave final approval of the version to be published.

## Pre-publication history

The pre-publication history for this paper can be accessed here:

http://www.biomedcentral.com/1471-2458/10/777/prepub

## Supplementary Material

Additional file 1 **"HIV, HBV and HCV incidence studies"**. Click here for file

Additional file 2 **"HIV, HBV and HCV studies by infection and risk group"**. Click here for file
